# Lake Bacterial Assemblage Composition Is Sensitive to Biological Disturbance Caused by an Invasive Filter Feeder

**DOI:** 10.1128/mSphere.00189-17

**Published:** 2017-05-31

**Authors:** Vincent J. Denef, Hunter J. Carrick, Joann Cavaletto, Edna Chiang, Thomas H. Johengen, Henry A. Vanderploeg

**Affiliations:** a Department of Ecology and Evolutionary Biology, University of Michigan, Ann Arbor, Michigan, USA; b Department of Biology, Central Michigan University, Mount Pleasant, Michigan, USA; c NOAA Great Lakes Environmental Research Laboratory, Ann Arbor, Michigan, USA; d Cooperative Institute for Limnology and Ecosystems Research, University of Michigan, Ann Arbor, Michigan, USA; University of Illinois at Urbana-Champaign

**Keywords:** 16S rRNA gene, invasive species, disturbance, freshwater ecology, microbial ecology, quagga mussel

## Abstract

One approach to improve forecasts of how global change will affect ecosystem processes is to better understand how anthropogenic disturbances alter bacterial assemblages that drive biogeochemical cycles. Species invasions are important contributors to global change, but their impacts on bacterial community ecology are rarely investigated. Here, we studied direct impacts of invasive dreissenid mussels (IDMs), one of many invasive filter feeders, on freshwater lake bacterioplankton. We demonstrated that direct effects of IDMs reduced bacterial abundance and altered assemblage composition by preferentially removing larger and particle-associated bacteria. While this increased the relative abundances of many free-living bacterial taxa, some were susceptible to filter feeding, in line with efficient removal of phytoplankton cells of <2 μm. This selective removal of particle-associated and larger bacteria by IDMs altered inferred bacterial functional group representation, defined by carbon and energy source utilization. Specifically, we inferred an increased relative abundance of chemoorganoheterotrophs predicted to be capable of rhodopsin-dependent energy generation. In contrast to the few previous studies that have focused on the longer-term combined direct and indirect effects of IDMs on bacterioplankton, our study showed that IDMs act directly as a biological disturbance to which freshwater bacterial assemblages are sensitive. The negative impacts on particle-associated bacteria, which have been shown to be more active than free-living bacteria, and the inferred shifts in functional group representation raise the possibility that IDMs may directly alter bacterially mediated ecosystem functions.

**IMPORTANCE** Freshwater bacteria play fundamental roles in global elemental cycling and are an intrinsic part of local food webs. Human activities are altering freshwater environments, and much has been learned regarding the sensitivity of bacterial assemblages to a variety of these disturbances. Yet, relatively few studies have focused on how species invasion, which is one of the most important aspects of anthropogenic global change, affects freshwater bacterial assemblages. This study focuses on the impact of invasive dreissenid mussels (IDMs), a globally distributed group of invasive species with large impacts on freshwater phyto- and zooplankton assemblages. We show that IDMs have direct effects on lake bacterioplankton abundance, taxonomic composition, and inferred bacterial functional group representation.

## Abstract (precis)

Freshwater bacteria play fundamental roles in global elemental cycling and are an intrinsic part of local food webs. Human activities are altering freshwater environments, and much has been learned regarding the sensitivity of bacterial assemblages to a variety of these disturbances. Yet, relatively few studies have focused on how species invasion, which is one of the most important aspects of anthropogenic global change, affects freshwater bacterial assemblages. This study focuses on the impact of invasive dreissenid mussels (IDMs), a globally distributed group of invasive species with large impacts on freshwater phyto- and zooplankton assemblages. We show that IDMs have direct effects on lake bacterioplankton abundance, taxonomic composition, and inferred bacterial functional group representation.

## INTRODUCTION

Aquatic bacteria play key roles in nutrient cycling and contribute significantly to global biomass and energy fluxes (1, 2). For example, bacterial respiration of terrestrial carbon subsidies contributes to global net freshwater CO_2_ emissions (2 Pg/year) that rival net uptake by the oceans (2.6 Pg/year) despite the relatively small footprint of freshwater systems (3). Anthropogenic disturbances can alter bacterial assemblages, and these changes in composition can either mitigate the predicted direct effects of these disturbances (4, 5) or lead to major shifts in bacterially mediated fluxes (6, 7). As such, understanding links between global change and bacterial assemblages is essential for predicting what our future biosphere will look like.

Species invasion is one of the three main components of global change (8) and provides a particularly useful phenomenon to help address knowledge gaps in microbial disturbance ecology (9) as we can readily determine (i) the initial response of species introduction in laboratory or field experiments and (ii) the *in situ* long-term response to this persistent disturbance by comparing systems that have and have not been invaded. In this study, we focused on the initial response to direct impacts by filter-feeding invasive dreissenid mussels (IDMs)—specifically *Dreissena bugensis* (quagga mussel). IDMs are compelling as (i) they are among many filter feeders that have invaded freshwater and coastal marine systems (e.g., Asian carp, golden mussel, and Asian clam) (10, 11), (ii) they continue to spread worldwide, including to over 30 U.S. states (10), and (iii) their high filtering rates and densities of >10,000 individuals/m^2^ have led to ecosystem restructuring of both pelagic and benthic environments (10, 12).

The impacts of IDMs and invasive species more generally on a range of aquatic organism have been well documented (10); however, their impacts on bacterial assemblages have received limited attention. Direct negative effects on bacterial abundance by filter feeding have only been observed in experiments performed under meso- to eutrophic conditions (13–15). *In situ* changes in bacterial abundance following IDM invasion have instead been attributed to indirect effects through IDM-mediated reduction of nanozooplankton bacterial predators (16). Studies of effects on bacterial assemblage composition have focused on bacteria involved in primary production, particularly the induction of cyanobacterial blooms through preferential feeding on eukaryotic phytoplankton (17, 18). Other bacterial functional groups have also been considered only in studies of indirect effects on bacterial assemblage composition (14, 15, 19, 20). Thus, most studies have not identified the direct effects of IDMs. In addition, most previous studies did not resolve impact at a taxonomic level beyond the phylum or class level and have not inferred any impacts on bacterially mediated processes, creating a knowledge gap that limits our understanding of the ecosystem impacts of IDM.

Here, we contribute to our understanding of bacterial responses to disturbance by determining how IDMs shape lake bacterioplankton assemblages. We performed short-term laboratory experiments using Lake Michigan water and IDMs to accomplish a realistic test of the response of native plankton communities to IDMs at *in situ* mussel densities. We specifically determined direct IDM impacts on lake bacterial abundance and assemblage composition. We also tracked IDM impacts on bacteriovorous zooplankton to verify the absence of indirect food web effects. To test if selective filtering of larger and particle-associated (PA) bacteria explained observed assemblage composition shifts, we compared taxon-level IDM impacts to taxon-level preferences between free-living (FL) and PA lifestyles. Finally, we assessed whether IDM invasion may impact bacterial functional group representation by inferring functional groups based on energy and carbon source usage.

## RESULTS

We performed three laboratory mesocosm experiments in a 2-year period to test the direct effects of IDMs on bacterial abundance and assemblage composition. We used water and IDMs collected from Lake Michigan and assessed filtering rates in all experiments by tracking changes in chlorophyll levels, while plankton counts were tracked for one of the experiments as well.

### IDM impacts on plankton abundance.

Based on chlorophyll *a* (Chl *a*) removal rates as a measure of phytoplankton abundance, higher feeding rates were observed in the experiments performed in July 2013 and August 2014 (~50% Chl *a* removal), compared to December 2014 (23.3%) (Table 1). For the experiment where phytoplankton cell counts were determined (August 2014), Chl *a* removal rates corresponded well with reductions in phytoplankton populations (Table 1). Chl *a* removal rates in the 0.7- to 2-μm fraction were similar to those in the >2-μm fractions (Table 1). For the August 2014 experiment, we also examined bacterioplankton and zooplankton population changes. Mussel feeding removed ~14% of bacterial cells by the end of the experiment (Table 1). Counts of small ciliated and flagellated zooplankton indicated active growth during the experiment and removal of both groups in the presence of IDMs (Table 1; see Table S1 in the supplemental material).

10.1128/mSphere.00189-17.3 TABLE S1  Quantification of phytoplankton and zooplankton populations. The average abundance and standard deviation across replicates are given across taxonomic groups grouped as “diatoms,” “other algae,” “ciliates,” and “HNFs” in Table 1. Download TABLE S1, PDF file, 0.1 MB. Copyright © 2017 Denef et al. 2017 Denef et al. This content is distributed under the terms of the Creative Commons Attribution 4.0 International license.

**TABLE 1:** Impact of IDM feeding on plankton populations^a^

Date and fraction	Amt of Chl *a* or cells (μg or cells/liter)			Removal %
	Initial	Final control	Final IDMs	
25 July 2013				
Chl *a*				
0.7–2 μm	0.19	0.16	0.06 (0.01)	60.5 (13.0)
2–153 μm	0.83	0.79	0.42 (0.13)	47.2 (14.7)
Total	1.01	0.95	0.48 (0.14)	49.5 (14.4)
15 August 2014				
Chl *a*				
0.7–2 μm	0.19	0.19	0.10 (0.01)	48.1 (6.2)
2–20 μm	0.14	0.18	0.07 (0.02)	63.1 (19.1)
20–153 μm	0.04	0.05	0.03 (0.01)	37 (11.2)
Total	0.37	0.41	0.19 (0.03)	53.3 (8.8)
Diatoms (total)	9.5E+03 (1.4E+03)	11.4E+03 (3.8E+03)	6.8E+03 (4.6E+03)	40.8 (53.5)
Other algae (total)	3.0E+05 (1.1E+05)	4.6E+05 (0.7E+05)	1.7E+05 (0.4E+05)	62.5 (19.8)
Ciliates (total)	7.4E+02 (2.2E+02)	8.2E+02 (5.6E+02)	4.0E+02 (2.6E+02)	51.4 (83.3)
HNFs (total)	1.0E+06 (0.2E+06)	1.5E+06 (0.06E+06)	1.2E+06 (0.2E+06)	19.7 (11.8)
Bacterial cells (total)	4.9E+08	4.7E+08 (0.4E+08)	4.0E+08 (0.4E+08)	14.4 (11.2)
19 December 2014				
Chl *a*				
0.7–2 μm	0.22	0.19	0.16 (0.01)	14.3 (1.3)
2–20 μm	0.09	0.02	0.03 (0.00)	−8.1 (0.8)
20–153 μm	0.03	0.13	0.09 (0.03)	32.3 (9.5)
Total	0.34	0.34	0.28 (0.02)	19.6 (1.6)

a The percentage of phytoplankton removed was determined based on Chl *a* in all experiments, while bacterioplankton, phytoplankton, and zooplankton cell counts were only quantified for the August 2014 experiment. Removal percentages were calculated by subtracting final concentrations in the presence of IDMs from those in the no-IDM control and dividing by the final concentrations in the no-IDM control: a negative value indicates increase. Numbers in parentheses indicate standard deviations. Bacterial cell numbers were equated to total cells counted after DAPI staining, which in Lake Michigan are composed for >99% of bacteria (24). HNFs, heterotrophic nanoflagellates.

### IDM impacts on bacterioplankton composition.

**FIG 1:**
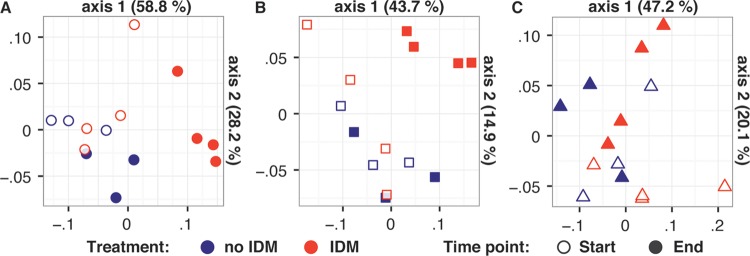
Direct impacts of IDM feeding on bacterial assemblage composition. Shown are results from principal-coordinate analysis (PCoA) ordinations of bacterial assemblage composition at the start and end of the mussel filter feeding experiments performed in (A) July 2013, (B) August 2014, and (C) December 2014.

At the end of the experiment, we observed a small, albeit significant effect of mussel addition after consideration of variance explained by experimental date (permutational multivariate analysis of variance [PERMANOVA], *R*^2^ = 0.04, *P* = 0.002) (Fig. 1). When analyzing only the two experiments with high feeding rates (July 2013 and August 2014), slightly more of the assemblage composition variance was explained by mussel presence/absence (PERMANOVA, *R*^2^ = 0.08, *P* = 0.004). Analysis of molecular variance (AMOVA) allowed us to explore these results in more detail by testing the significance of each pairwise comparison between treatments. For both high-feeding-rate experiments, bacterial assemblage composition was significantly different after IDM feeding compared to before and also between mussel-free controls and IDM treatments at the end of the experiment (Table 2). In contrast, the experiment with lower feeding rates (December 2014) did not reveal significant effects of IDM presence on bacterial assemblage composition (AMOVA) (Table 2). For all three experiments, assemblage composition for no-IDM controls did not differ between controls and mussel-added treatments at the start of the experiment (Table 2). Finally, bacterial assemblage composition was significantly different in July 2013 and August 2014, but not December 2014 when comparing treatments with IDM added at the end of the experiment to all other treatments (Table 2). These test data supported the observed clustering patterns on the principal coordinate ordination (Fig. 1A to C).

**TABLE 2:** Significant shifts in bacterial assemblage composition due to IDM presence when feeding rates were high

Date	Shift in assemblage composition for^a^:				
	C_s_ vs M_s_	C_e_ vs C_s_	M_e_ vs M_s_	M_e_ vs C_e_	M_e_ vs C_s_ + C_e_ + M_s_
July 2013	0.40	0.20	**0.02**	**0.02**	**0.001**
August 2014	0.88	0.28	**0.03**	**0.04**	**0.003**
December 2014	0.39	0.51	0.15	0.09	0.15

a Shown are *P* values of pairwise AMOVA comparisons to test for significant differences (highlighted in boldface) in bacterial assemblage composition. C, control; M, IDM added. The subscripts “s” and “e” refer to the start (C_s_ and M_s_) and end (C_e_ and M_e_) of the experiment.

**FIG 2:**
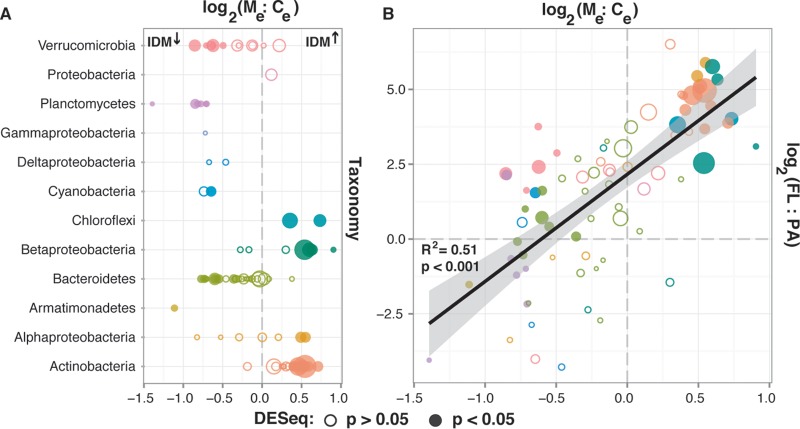
Impacts of IDMs at taxonomic level due to differential impact on FL and PA taxa. (A) Impact of assemblage shifts due to filter feeding on bacterial taxonomic groups (July 2013 and August 2014 experiments only). The log_2_ values of the ratios of relative abundances in the mussel treatment (M_e_) relative to the control treatment (C_e_) at the end of the experiment were plotted for OTUs with an average relative abundance of >0.1% across all experimental samples. Circle size was scaled based on average relative abundance; filled circles indicate OTUs with significantly higher (IDM^↑^) or lower (IDM^↓^) relative abundance when IDMs were present. (B) Correlation between OTU sensitivity to filter feeding (log_2_ ratio of M_e_ to C_e_) and differential relative abundance of OTUs between PA and FL fractions (log_2_ ratio of FL to PA). Data on relative abundance differences between FL (0.22- to 3-μm) and PA (>3-μm) fractions were derived from fractionated August 2014 experiment water and fractionated Lake Michigan field samples collected at nearby stations 10 days before the water for the July 2013 experiment was collected (Fig. S2).

We identified bacterial taxa that changed in relative abundance as a result of mussel presence by analyzing the July 2013 and August 2014 data using DESeq2, a method that identifies those taxa that have significantly different relative abundances between treatments. Taxa belonging to the *Chloroflexi* (CL500-11), *Actinobacteria* (several acI lineages), *Alphaproteobacteria* (LD12 and alfI-A1), and *Betaproteobacteria* (BetI-A, PnecB, and LD28) increased in relative abundance, while taxa belonging to the *Bacteroidetes* (bacII, bacV, and bacVI), *Verrucomicrobia* (*Opitutae*, *Spartobacteria*, and OPB35), *Cyanobacteria*, *Planctomycetes* (CL500-3), and *Armatimonadetes* (*Armatimonas*) decreased in relative abundance (*P* < 0.05) (Fig. 2A; see Table S2 in the supplemental material). This led to a further increase in relative abundance of groups that already were highly dominant in the community such as the betI-A, acI-B1, and acI-A6 lineages. These shifts caused inferred bacterial functional groups to change in relative abundance as well, most prominently a reduced relative abundance of autotrophic phototrophs (Chl *a*) and increased relative abundance of heterotrophic chemoorganotrophs predicted to be capable of rhodopsin-dependent phototrophic energy generation (see Fig. S1A and Table S2 in the supplemental material).

10.1128/mSphere.00189-17.1 FIG S1  Impacts of IDMs at the functional group level due to differential impacts on FL and PA taxa. (A) Impact of assemblage shifts due to filter feeding on bacterial functional group representation (July 2013 and August 2014 only). The log_2_ values of the ratios of relative abundances in the mussel treatment (M_e_) relative to the control treatment (C_e_) at the end of the experiment were plotted for OTUs with an average relative abundance of >0.1% across all experimental samples. Circle size was scaled based on average relative abundance: filled circles indicate OTUs with significantly higher (IDM^↑^) or lower (IDM^↓^) relative abundance when IDMs were present. The *y* axis groups OTUs into inferred functional groups based on energy source usage, while color groups OTUs based on carbon source usage (heterotroph, autotroph, or mixotroph). Organo, chemoorganotroph; Photo, phototroph (subscripts: chl, Chl *a*; bc, bacteriochlorophyll; rh, rhodopsin); Litho, chemolithotroph (subscripts: S, sulfur; N, NH_4_^+^). Some OTUs were predicted to have multiple metabolic lifestyles, and these are indicated by the bottom 4 categories: e.g., Organo/Photo_bc_. (B) Correlation between OTU sensitivity to filter feeding (log_2_ ratio of M_e_ to C_e_) and differential relative abundance of OTUs between PA and FL fractions (log_2_ ratio of FL to PA). Data on relative abundance differences between FL (0.22 to 3 μm) and PA fractions (>3 μm) were derived from fractionated August 2014 experiment water and fractionated Lake Michigan field samples collected at nearby stations 10 days before the water for the July 2013 experiment was collected (Fig. S2). Download FIG S1, PDF file, 0.1 MB. Copyright © 2017 Denef et al. 2017 Denef et al. This content is distributed under the terms of the Creative Commons Attribution 4.0 International license.

10.1128/mSphere.00189-17.4 TABLE S2  Classification and average and differential relative abundances of the most abundant OTUs. We used the DESeq2 R package to determine significant differences in relative abundance of OTUs with an average abundance of >0.1% across samples as a function of experimental treatment, habitat preference (PA versus FL), and inland lake invasion status. *P* values were adjusted for multiple testing with the Benjamini-Hochberg false discovery rate correction. Selection of the most abundant OTUs prior to DESeq2 analysis was performed independently for feeding experiments, comparison of PA versus FL habitats, and the inland lake survey. RHO, rhodopsin; CHL, chlorophyll *a*; BCHL, bacteriochlorophyll. Download TABLE S2, PDF file, 0.1 MB. Copyright © 2017 Denef et al. 2017 Denef et al. This content is distributed under the terms of the Creative Commons Attribution 4.0 International license.

We compared the changes in relative abundance of bacterial taxa between the end and start of the experiments to differences in relative abundance of the same taxa between free-living (FL) and particle-associated (PA) habitats. FL and PA habitats were operationally defined based on cells passing through or being retained on a 3-μm-pore-size filter, respectively. Most taxa that increased in relative abundance after experimental mussel feeding had higher relative abundances in the FL fraction, while most taxa that decreased in relative abundance after feeding had higher relative abundances in the PA fraction (Fig. 2B). Yet, some operational taxonomic units (OTUs) that had higher relative abundance in the FL than PA fraction were found at lower relative abundance after exposure to IDMs (left upper quadrant in Fig. 2B [e.g., *Verrucomicrobia*]).

For the analysis of FL versus PA preference, we included fractionated samples from the Lake Michigan experimental water used in August 2014, and as the experimental water had not been fractionated in 2013, Lake Michigan field samples taken 10 days prior to the July 2013 experiment. As no field samples were available from the exact location where the experimental water was sampled in 2013 (40 m below the water surface at the 45-m depth station), we used data from two stations where both filter fractions were available and the water conditions and assemblage composition most closely resembled those of the experimental samples (35 m below the water surface at the 110-m depth station and 10 m below the water surface at the 15-m depth station) (see Fig. S2A in the supplemental material). Despite sample location and methodological differences (see Materials and Methods), we observed high correlation levels between the OTU FL/PA ratios in the field (July 2013) and experimental (August 2014) data (Fig. S2B).

10.1128/mSphere.00189-17.2 FIG S2  Evaluation of the validity of using fractionated data from July 2013 field samples in conjunction with experimental data from July 2013 and August 2014. We assessed whether use of both 2013 and 2014 summer fractionated data would not be negatively impacted by differences between 2013 field samples and experimental data based on (i) sampling locations in Lake Michigan, (ii) particle-associated fraction considered, (iii) DNA extraction protocol, and (iv) differences in sequencing facilities generating the data. (A) PCoA ordination of experimental samples (collected at 5 m above the bottom at the 45-m depth station) and field samples collected in July 2013. Sequence libraries for PA assemblages from the deep offshore waters (110 m [bottom]) failed to amplify and hence could not be included in the analysis of fraction preferences. Samples from 110 m (Chl *a* max) and 15 m (bottom) were used for analysis of OTU differential relative abundances between PA and FL fractions (marked with asterisks). Note that the FL field sample bacterial assemblages (filled circles) are expected to be more similar to unfractionated samples (as used for the experiment) than PA field samples (open circles), as the FL fraction contains the majority of bacterial cells. (B) Correlation between FL/PA ratios from (*x* axis) the 2014 experimental water sample, fractionated in 0.22- to 3-μm and 3- to 153-μm fractions, extracted using the MoBio PowerWater extraction kit, and sequenced at the University of Michigan and (*y* axis) the 2013 field water samples, fractionated in 0.22- to 3-μm and 3- to 20-μm fractions, extracted using the Qiagen AllPrep Universal kit, and sequenced by the Joint Genome Institute. Despite the differences in methods and times/locations of sampling, the FL/PA relative abundance ratios of OTUs showed high correspondence. Data points are scaled based on their average relative abundance in the experimental samples (i.e., 0.22- to 153-μm fractions used in July 2013, August 2014, and December 2014), and filled in case there was a significant differential representation between FL and PA fractions in the August 2014 experimental water. Download FIG S2, PDF file, 0.1 MB. Copyright © 2017 Denef et al. 2017 Denef et al. This content is distributed under the terms of the Creative Commons Attribution 4.0 International license.

## DISCUSSION

Thus far, reports of impacts by invasive dreissenid mussels (IDMs) on freshwater plankton assemblages have focused primarily on the decimation of phyto- and zooplankton densities (10, 21) and shifts in their composition (12, 22–24). Bacterioplankton assemblages have received much less attention. This bias is likely due to methodological limitations to identify bacteria *in situ* at the time when interest in IDM impacts on freshwater biota peaked following their arrival to North America in the late 1980s. Our work shows that IDMs can have direct effects on lake bacterioplankton abundance and composition. Understanding how a globally distributed invasive species is reshaping freshwater bacterial assemblages is important as bacteria play fundamental roles in freshwater community ecology (1) and ecosystem functioning (25). While many bacterial assemblage composition shifts may not have system-level functional consequences, disturbance-induced changes to assemblage composition do often lead to shifts in elemental fluxes (9), including system-level greenhouse gas emissions (7).

IDMs have been shown to feed on a broad range of particles and to exhibit taxon-specific selective feeding within different phyto- and zooplankton size ranges (23, 26). In Lake Michigan, experiments and modeling have shown that clearance rates and mussel abundances in the mid-depth region (~30 to 70 m) were sufficiently high to decimate the spring phytoplankton bloom based on feeding rates in <53- and >53-μm size fractions of Chl *a* (21, 27). It surprised us to find that removal rates of picophytoplankton (0.7 to 2 μm) were similar to those for both nano- and microphytoplankton as this extends IDM impacts to even the smallest fractions of photosynthetic organisms in Lake Michigan. This study extends to oligotrophic environments the findings from a previous study on a eutrophic lake that indicated similar feeding rates on phytoplankton sized <1 and >30 μm (26).

In addition to picophytoplankton, we observed that IDMs removed bacteria from the water column. Previous laboratory experiments have shown that zebra mussels can remove bacterium-size (1-μm) synthetic particles at rates up to 37% of total phytoplankton removal rates (13) and can filter laboratory-cultured bacteria 1.3 to 4.1 μm in size (28). Our results here are important, because *in situ*, direct grazing effects on bacterial abundance were thought to be restricted to meso- and eutrophic lakes with high bacterial densities (13, 15). Similarly, in the Hudson River, less than 2% of dreissenid mussel growth was attributed to ingestion of free-living bacteria (14). As the Lake Michigan water used for the experiment was nutrient poor, we had expected limited direct feeding effects. Yet, we observed high bacterial removal during our experiment. As the removal rates were approximately 3 orders of magnitude higher than what has been observed as a result of micro- and nanozooplankton grazing (29), we attributed this removal primarily to IDM feeding activity.

We hypothesized that these high bacterial cell removal rates were attributable to the removal of large-size cells and particle-associated (PA) bacteria. As PA assemblages have been shown to significantly differ in composition from free-living (FL) assemblages, even at phylum- to class-level taxonomic resolution (30–34), we predicted that this selective removal would explain the observed assemblage composition shifts. Our data supported this hypothesis. In light of this observation, it has to be noted that our automated microscopic counts may have counted some clusters of PA bacteria as single cells, and thus our feeding rates may have been underestimated. Yet, from the relationship between taxon-specific shifts in relative abundance due to IDM feeding and taxon-specific relative abundance differences between PA and FL fractions, it was clear that a significant fraction of cells that passed through the 3-μm filter (which is our and several previous studies’ operational definition for the PA bacterial fraction [32, 34, 35]) were removed as well. This is in line with our observation of similar Chl *a* removal rates for the 0.7- to 2-μm and >2-μm fractions.

An alternative hypothesis that we evaluated is that indirect food web effects might have contributed to shifts in bacterial assemblage composition. Even though we designed the experiment to measure direct mussel feeding effects by limiting its duration (<4 h), mussel removal of microzooplankton seen here and in previous reports (22) can relieve top-down control on heterotrophic nanoflagellates (HNFs) (36), which are their preferred food (37). Therefore, because HNFs are the primary consumers of bacterioplankton (38), IDMs could have indirectly increased bacterivory by HNFs through a simple trophic cascade, resulting in decreased bacterial numbers and assemblage composition shifts. Having said that, the numbers of both HNFs and ciliates were negatively affected by IDMs compared to controls. Also, considering no impact on bacterial assemblage composition was observed in control treatments, microzooplankton feeding was unlikely to have had a strong impact on bacterial assemblage composition relative to much higher IDM feeding rates. This again is in line with the much lower reported grazing rates of microzooplankton on bacteria compared to our observed rates (29).

A final alternate hypothesis we could test is that experimental assemblage composition shifts could have been caused by release of IDM-associated bacteria. During the experiment, IDMs actively fed on plankton and excreted (pseudo)feces (23). Except for a few *Betaproteobacteria* taxa typical of lake water column environments, the classes and phyla that predominate in the mussel microbiome (*Gammaproteobacteria*, *Planctomycetes*, and *Betaproteobacteria* [14, 39]) did not increase in relative abundance during our experiment.

As we noted a decrease in total bacterial cell numbers due to IDM feeding, an increase in relative abundance of a taxon indicated a higher ability to escape from filter feeding relative to taxa that remained constant or decreased in relative abundance during the experiment. IDM feeding thus led to the relative enrichment of taxa at a class to phylum level as well as at the level of inferred functional groups. The fact that cell numbers did not increase in the controls further supports our inference that the increase in relative abundance of certain taxa was due to maintenance of their population size due to feeding resistance while other taxa declined rather than due to active growth. A case in point is the acI *Actinobacteria*; their abundance in freshwater lakes has been attributed to their small cell size and cell envelope characteristics, which allow them to escape nanozooplankton grazing (40). These traits may also explain their relatively high resistance to IDM feeding. A few FL taxa known to have cell sizes in the upper range of the 0.22- to 3-μm size fraction, particularly *Chloroflexi* CL500-11 (41, 42), increased in relative abundance after feeding, contrary to our expectations. Hence, their enrichment during the experiment may indicate traits that protect this organism from grazing and these traits may explain their predominance (up to 50% of all cells) in the hypolimnion of deep lakes around the world (41). In line with the decrease in relative abundance of larger and PA bacteria after feeding, larger, fast-growing cells have been shown to serve as preferential food sources for microzooplankton in aquatic systems (43).

Previous studies have shown bacterial abundance and assemblage shifts due to the long-term presence of invasive filter feeders (14, 19, 44). These studies could not disentangle direct versus indirect effects, such as stimulation by (pseudo)feces or complex food web interactions. The only study that identified specific taxa affected by the presence of IDMs showed enrichment for *Gammaproteobacteria* and *Betaproteobacteria* and a reduced relative abundance of *Deltaproteobacteria* and *Flavobacteria* relative to background levels (14). We did observe similar negative effects on *Flavobacteria* and other *Bacteroidetes* and enrichment of several *Betaproteobacteria* taxa, but no significant effect on *Gammaproteobacteria* and *Deltaproteobacteria*. Precise comparison to our results is difficult since this previous study used oligonucleotide probes that missed 1/3 of metabolically active populations, and their data were derived from a river system, which harbors significantly different bacterial assemblages than lakes (45).

Literature-based inferences of OTU-level energy and carbon source usage (Table S2; similar to reference 46) indicated impacts on the relative representation of inferred bacterial functional groups during the experiment. Trait inferences from 16S rRNA gene data are challenging due to variable phylogenetic trait conservation (47). Thus, these broad inferences at the level of C and energy source are limited in both the confidence of the functional group assignment and our ability to draw inferences from these broad groupings to impacts on system functioning. The confidence level of functional group assignment was helped by the meticulous work that has been done to classify freshwater taxa at so-called “tribe” levels and the freshwater microbiology research community’s adherence to these classifications when describing isolate genomes, physiological assays, or sequencing surveys (48). We took a conservative approach in that we assigned taxa to the “heterotrophic chemoorganotroph” functional group unless tribes were consistently classified otherwise. As available data are limited at this point, particularly regarding chemolithotrophic energy metabolism and autrophic growth capabilities, our analysis lacked insights as far as the impact on these latter metabolisms was concerned. Despite these limitations, we are confident about the main shift that was inferred, which was a strong increase in the predominance of heterotrophic chemoorganotrophs predicted to be capable of energy generation using rhodopsin. Genomic analyses of the affected taxonomic groups inferred to have these metabolic traits (predominantly acI *Actinobacteria*, but also LD12 and CL500-11) are consistently retrieving the genes encoding these traits (42, 49, 50).

Our study demonstrates direct impacts that invasive filter feeders, specifically IDMs, can have on water column bacterial assemblages and emphasizes the strong impacts on PA bacteria. Considering the large differences that exist between FL and PA freshwater bacterial assemblages (31, 32, 34) and the disproportionately high activities of PA bacteria (51), their specific removal makes functional implications from direct invasive filter feeding impacts likely. While further evaluation of impacts on microbially mediated functions is needed (by measuring elemental fluxes and/or omics analyses), as well as of how these direct short-term effects translate into longer-term effects, our study highlights the potential of previously unrecognized impacts of IDM on aquatic systems through changes to bacterioplankton assemblages. If we are to improve models that address how global change affects bacterial assemblages and ecosystem functions they mediate (52), we must better understand and integrate bacterial responses to all types of disturbances, including those to species invasions.

## MATERIALS AND METHODS

### Feeding experiment design.

Quagga mussels (lake floor) and lake water (5 m above lake floor) were collected from a 45-m water column depth station in Lake Michigan offshore of Muskegon, MI (43°12′N, 86°27′W), on 24 July 2013, 14 August 2014, and 19 December 2014 and were maintained at lake temperature (5 to 7°C) during transport (<8 h) and experimentation. This location is at the depth range where mussel concentrations and filtering impacts are highest (21). In the lab, mussels were cleaned of debris and placed in a tank filled with 90 liters of 153-μm-screened (to remove grazing mesozooplankton [21, 23]) Lake Michigan water. The next morning, we transferred mussels to a 40-liter aquarium with 153-µm-screened Lake Michigan water for 2 h. The mussel cleaning and ~14-h reacclimation period removed external periphyton and debris, cleared mussel guts of sediment ingested during capture, and gave the mussels time to reach digestive equilibrium with their natural food source. All materials were washed with bleach and rinsed with deionized water to minimize bacterial contamination. Seven 19-liter high-density polyethylene (HDPE) buckets were filled with 16 liters of 153-µm-screened lake water each. Fifteen adult mussels with an average (±standard deviation [SD]) length of 22.2 ± 0.9 mm were added to each of four buckets, and three buckets remained mussel free. Gentle mixing was provided by bubbling air through a pipette. Water samples were taken prior to the addition of mussels and again 3 to 3.7 h after mussels had opened up, a sign of active feeding (~15 min after adding them to the buckets). The number of mussels added and experiment duration were chosen to allow healthy mussels to clear 30 to 60% of preferred seston (21). The experiment was limited to ~3.5 h to minimize indirect effects of mussel nutrient excretion on phytoplankton growth and the removal of microzooplankton grazers that could lead to underestimates of the feeding on some plankton (53).

### Assessment of impacts on plankton abundance.

Size-fractionated chlorophyll *a* (Chl *a*) was analyzed by filtering water through stacked 47-mm-diameter filters: a 20-µm-pore-size Nitex screen (if used), a 2-µm Isopore TTTP filter (Millipore), and a GF/F filter (nominal pore size, 0.7 µm [Whatman]) (17), resulting in 20- to 153-µm, 2- to 20-µm, and 0.7- to 2-µm size categories. Total Chl *a* was calculated as the sum of the size categories. Screens and filters were inserted into plastic test tubes, frozen, extracted with *N*,*N*-dimethylformamide, and analyzed fluorometrically (54).

For the August 2014 experiment only, we measured the abundance of bacterioplankton, nanoplankton, and microplankton in triplicate subsamples of water collected at the start (for 2 control buckets and 2 buckets with mussels added) and end (all seven buckets) of the experiment; these were fixed in 1% (wt/vol) paraformaldehyde, 1% (wt/vol) glutaraldehyde, and 2% Lugol’s solution, respectively. All samples were subsequently stored at 4°C. For bacterioplankton, we concentrated fixed water samples on 0.22-μm-pore-size polycarbonate filters (Millipore) and examined DAPI (4′,6-diamidino-2-phenylindole)-stained filters using a Zeiss AxioImager M2 epifluorescence microscope for cell abundance. A minimum of 2,000 DAPI-stained cells across 15 fields of view at ×1,000 magnification were counted per filter. Nanoplankton cells (2 to 20 µm) were concentrated onto 0.8-µm-pore-size polycarbonate filters (Poretics) and stained with primulin (37). Cells in random fields were counted until a total of 200 to 300 cells was reached using a Leica DMR 5000 (Wetzlar, Germany) epifluorescence microscope (×1,000 magnification). Dominant pigment fluorescence (UV, Chl *a*, phycobilin proteins) of individual cells and their cellular morphology were used to make taxonomic determinations. Microplankton cells (20 to 200 µm) were dispensed into settling chambers (80- to 100-ml volume) and were allowed to settle for 24 h onto coverslips (37). All cells present in each chamber (~400 to 500 cells) were enumerated using an inverted microscope (Leica DMI 4000) at ×200 magnification. Nano- and microplankton cells were enumerated to their lowest taxonomic position (55).

### Impacts on bacterioplankton assemblage composition.

One liter was sampled from all buckets at the start and end of the experiment for bacterial assemblage composition analysis. Immediately after sampling, bacteria were collected on 0.22-μm Sterivex columns (Millipore) using a peristaltic pump (~100 ml/min). In addition, three replicate 1-liter samples taken at the start of the August 2014 experiment were fractionated in 3- to 153-μm and 0.22- to 3-μm fractions by sequential filtering on a 3.0-μm polycarbonate filter (Millipore) and 0.22-μm Sterivex column. As no fractionated samples were generated for the other experiment with high feeding rates (July 2013), we made use of fractionated (0.22- to 3-μm and 3- to 20-μm) field survey samples collected 9 to 10 days prior to the July 2013 experiment along the same nearshore to offshore transect that the experimental water originated from. This increased statistical power for FL (0.22- to 3-μm) to PA (>3-μm) comparisons. As this field survey did not include samples from the exact location that the experimental water was sampled from, we used two samples taken nearby with the least differences in physicochemical conditions and assemblage composition: 5 m above the lake floor, where the lake is 15 m deep (43°11′17″N, 86°20′38″W; 15 July 2013), and 35 m below the water surface, where the lake is 110 m deep (43°11′59″N, 86°34′11″W; 16 July 2013). Ten liters was sequentially filtered onto 142-mm 3.0-μm polycarbonate filters and 0.22-μm polyethersulfone filters (Millipore) (46). DNA from the feeding experiments was extracted using the PowerWater kit (MoBio), according to the manufacturer’s protocol, including the bead-beating step. DNA from the fractionated field samples was extracted using a modified AllPrep Universal kit protocol (Qiagen) (56).

### 16S rRNA gene sequencing and analysis.

We performed amplicon sequencing targeting the V4 region of the 16S rRNA gene (515F/806R) (57, 58) at the Joint Genome Institute (Lake Michigan field samples) and the University of Michigan Medical School (feeding experiments). Pooled libraries were sequenced on an Illumina MiSeq sequencer, using v2 chemistry 2 × 250 (500 cycles) paired-end reads. RTA v1.17.28 and MCS v2.2.0 were used to generate data. Analyses were performed with mothur v1.34.3 using the MiSeq standard operating protocol (accessed on 17 December 2014) for the generation of the operational taxonomic unit (OTU [97% sequence similarity]) table (59). Only bacterial sequences were retained. For classification, we used a hybrid protocol using a freshwater-specific taxonomy (48; https://github.com/mcmahon-uw/FWMFG) and the SILVA release 119 taxonomy (60).

Further analyses were carried out in R version 3.2.1 using phyloseq (61), vegan (62), and custom functions written by Michelle Berry (63). All figures were generated using the ggplot2 R package (64). Full code and input files are available at https://github.com/DenefLab/IDM_Experiments. All beta-diversity analyses were performed using Bray-Curtis dissimilarities based on OTU read count data scaled to the smallest library size (7,835 sequencing reads) (65). Differences in assemblage composition based on mussel presence/absence were determined with a nested permutational multivariate analysis of variance (PERMANOVA) (66) using the Adonis function (vegan) as well as by performing AMOVA analyses (67). We used the DESeq2 R package to determine significant differences in relative abundance of OTUs as a function of experimental treatment or habitat preference (PA versus FL) (65, 68). *P* values were adjusted for multiple testing through the Benjamini-Hochberg false discovery rate correction (68). We compared log_2_ ratios between analyses (habitat preference versus impact of mussels) by performing linear regressions with the lm function using ordinary least squares. For DESeq2 analyses, we only considered OTUs with an average abundance of >0.1% across samples considered in the different analyses. Selection of the most abundant OTUs prior to DESeq2 analysis was performed independently for feeding experiments and comparison of PA versus FL habitats. OTUs were also classified into functional groups based on carbon and energy source. We inferred membership to different functional classes based on a literature search (papers describing isolates, genome sequences and gene expression, or substrate utilization assays combined with fluorescent *in situ* hybridization) as described previously (46); the relevant references are listed in Table S2.

### Data availability.

Fastq files for Lake Michigan field samples are available through the Joint Genome Institute (http://genome.jgi.doe.gov/; project ID 1041198) and feeding experiment data through the NCBI sequence read archive (BioProject PRJNA385848).
