# Supplementary material for: Lake Bacterial Assemblage Composition Is Sensitive to Biological Disturbance Caused by an Invasive Filter Feeder
Source: mSphere. 2017 May 31;2(3):e00189-17. doi: 10.1128/mSphere.00189-17 (PMC5451517; doi:10.1128/mSphere.00189-17)
Supplement: TABLE S1 [file sph003172294st1.pdf]

Table S1

		No IDM control		IDM		
**Taxonomic Group**	**Description of Group**	**Initial Abundance (cells/mL)**	**Final Abundance (cells/mL)**	**Initial Abundance (cells/mL)**	**Final Abundance (cells/mL)**	**% removal (- means increase)**
**Bacillariophyta**	Diatoms	8.8 (1.8)	10.3 (3.3)	8.3 (1.3)	6.1 (4.2)	40.8 (53.5)
**Chorophyta**	Green algae	5.7 (4.9)	0 (0)	0 (0)	0 (0)	-
**Chrysophyta**	Yellow-brown algae	105.3 (19.7)	142.2 (21.5)	89.6 (26.5)	61.9 (23.5)	56.5 (24.0)
**Cryptophyta**	Red-brown algae	258.9 (82)	318.6 (48.5)	149.3 (71.2)	110.9 (51.7)	65.2 (24.4)
**Pyrrophyta**	Dinoflagellates	1 (0.2)	1.1 (0.5)	1 (0.2)	0.7 (0.3)	36.4 (55.6)

**Choanoflagellida**	Colorless, loricates	2.8 (4.9)	0 (0)	0 (0)	0 (0)	-
**Chrysomonadida**	Colorless, Chrysomonads	1172 (26.1)	1479.2 (85.9)	859.8 (95.4)	1164.9 (165)	21.2 (12.6)
**Cryptomonadida**	Colorless, Cryptomonads	5.7 (9.9)	42.7 (37.2)	0 (0)	57.6 (8.2)	-34.9 (94.3)
**Choreotrichida**	Ciliates	0.2 (0.1)	0.1 (0.1)	0.2 (0)	0.1 (0.1)	0 (0)
**Haptorida**	Raptorial Ciliates	0.1 (0)	0.1 (0)	0.1 (0.1)	0 (0)	100 (0)
**Oligotrichida**	Ciliates	0.1 (0.1)	0.2 (0.1)	0.1 (0)	0.1 (0)	50 (55.9)
**Prorodontida**	Ciliates	0.4 (0.3)	0.5 (0.4)	0.3 (0.1)	0.2 (0.2)	60 (101.5)
