# Supplementary figures and images for: Lake Bacterial Assemblage Composition Is Sensitive to Biological Disturbance Caused by an Invasive Filter Feeder
Source: mSphere. 2017 May 31;2(3):e00189-17. doi: 10.1128/mSphere.00189-17 (PMC5451517; doi:10.1128/mSphere.00189-17)

**Figure S1**

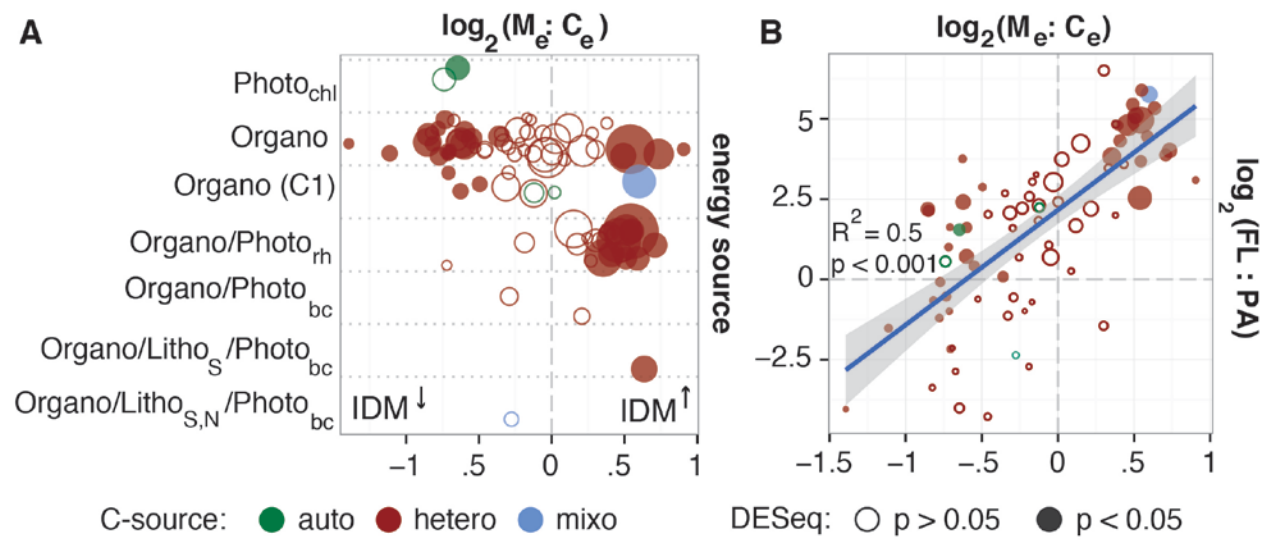

Supplement: FIG S1 [file sph003172294sf3.pdf]

**Figure S2**

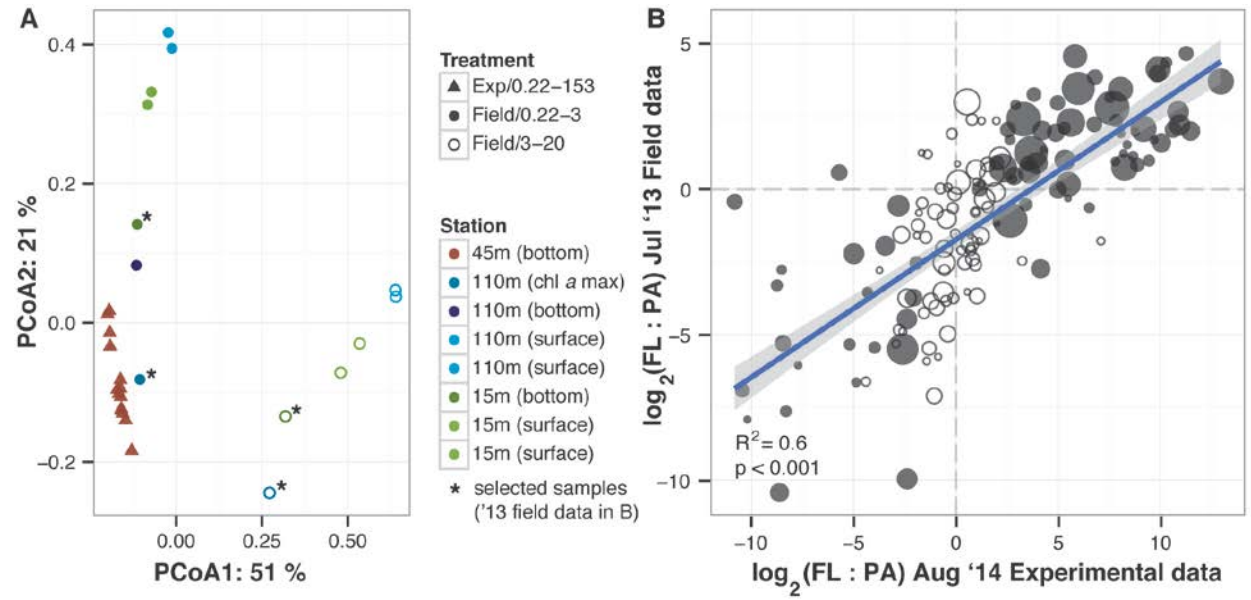

Supplement: FIG S2 [file sph003172294sf4.pdf]
