# Supplementary material for: Lake Bacterial Assemblage Composition Is Sensitive to Biological Disturbance Caused by an Invasive Filter Feeder
Source: mSphere. 2017 May 31;2(3):e00189-17. doi: 10.1128/mSphere.00189-17 (PMC5451517; doi:10.1128/mSphere.00189-17)
Supplement: TABLE S2 [file sph003172294st2.pdf]

Table S2

All datasets										July 2013 + August 2014 experiments			PA vs FL Aug14 Exp + Jul13 Field		
**OTU**	**Phylum**	**Class**	**Order**	**Family**	**Genus**	**Tribe**	**C_source**	**energy**	**Reference**	**log2(IDM:IDM-free)**	**DESeq2 p-value**	**mean relative abundance (%)**	**log2(FL:PA)**	**DESeq2 p-value**	**mean relative abundance (%)**
Otu00001	Actinobacteria	Actinobacteria	Actinomycetales	acI	acI-B	acI-B1	heterotroph	organo/photo(RHO)	Ghylin et al., 2014	0.54	0.00	12.65	4.97	0.00	6.40
Otu00002	Betaproteobacteria	Betaproteobacteria	Burkholderiales	betI	betI-A	unclassified	heterotroph	organo	Newton et al., 2011	0.54	0.00	8.37	2.54	0.00	5.62
Otu00003	Actinobacteria	Actinobacteria	Actinomycetales	acI	acI-A	acI-A6	heterotroph	organo/photo(RHO)	Ghylin et al., 2014	0.46	0.00	6.05	4.81	0.00	5.08
Otu00004	Bacteroidetes	Sphingobacteria	Sphingobacteriales	bacI	bacI-A	bacI-A1	heterotroph	organo	Newton et al., 2011; Woodhouse et al., 2016	-0.03	0.84	4.53	3.03	0.00	6.08
Otu00005	Cyanobacteria	Cyanobacteria	SubsectionI	FamilyI	unclassified	NA	autotroph	photo(CHL)	Boone and Castenholz, 2012	-0.65	0.00	0.92	1.55	0.00	2.75
Otu00006	Bacteroidetes	Sphingobacteria	Sphingobacteriales	bacIV	bacIV-B	Aquir	heterotroph	organo	Newton et al., 2011; Woodhouse et al., 2016	-	-	-	-2.17	0.00	0.79
Otu00007	Bacteroidetes	Flavobacteria	Flavobacteriales	bacV	unclassified	unclassified	heterotroph	organo	Newton et al., 2011	-0.05	0.72	2.83	0.69	0.12	6.31
Otu00008	Betaproteobacteria	Betaproteobacteria	Burkholderiales	betII	Pnec	PnecB	heterotroph	organo/litho(S)/photo(BCHL)	Livermore et al., 2014; Martinez-Garcia et al., 2012	0.63	0.00	1.18	5.33	0.00	1.30
Otu00009	Alphaproteobacteria	Alphaproteobacteria	Rickettsiales	alfV	alfV-A	LD12	heterotroph	organo/photo(RHO)	Newton et al., 2011	0.55	0.02	0.97	5.90	0.00	1.82
Otu00010	Actinobacteria	Actinobacteria	Actinomycetales	acI	acI-A	unclassified	heterotroph	organo/photo(RHO)	Ghylin et al., 2014	0.51	0.00	2.08	5.11	0.00	1.50
Otu00	Alphaproteob	Alphaproteob	Caulobacter	alfII	alfII-A	Brev	heterot	organo	Livermo	-0.83	0.06	0.19	-3.38	0.00	0.62

011	acteria	acteria	ales				roph		re et al., 2014						
Otu00012	Verrucomicrobia	Opitutae	Opitutales	Opitutaceae	unclassified	unclassified	heterotroph	organo(C1)	Kalyuzhnaya et al., 2008	-0.31	0.38	1.53	2.07	0.00	0.91
Otu00013	Betaproteobacteria	Betaproteobacteria	Burkholderiales	betI	betI-A	unclassified	heterotroph	organo	Newton et al., 2011	0.30	0.14	0.48	-1.45	0.01	0.85
Otu00014	Alphaproteobacteria	Alphaproteobacteria	Rhodobacterales	Rhodobacteraceae	unclassified	NA	heterotroph	organo/photo(BCHL)	Boone and Castenholz, 2012	-0.29	0.13	0.45	-0.56	0.05	0.56
Otu00015	Betaproteobacteria	Betaproteobacteria	Burkholderiales	betI	betI-A	Lhab-A1	mixotroph	organo/litho(S,NH4+)/photo(BCHL)	Zeng et al., 2012	-0.28	0.50	0.27	-2.37	0.00	0.55
Otu00016	Betaproteobacteria	Betaproteobacteria	Methylophilales	betIV	betIV-A	LD28	mixotroph	organo(C1)	Salcher et al., 2013	0.60	0.00	2.44	5.77	0.00	1.54
Otu00017	Bacteroidetes	Sphingobacteria	Sphingobacteriales	bacIII	bacIII-B	Algor	heterotroph	organo	Salcher et al., 2013; Woodhouse et al., 2016	0.03	0.84	1.82	3.74	0.00	2.70
Otu00018	Bacteroidetes	Sphingobacteria	Sphingobacteriales	bacIII	bacIII-A	unclassified	heterotroph	organo	Salcher et al., 2013	-0.23	0.14	1.13	2.22	0.00	2.27
Otu00019	Betaproteobacteria	Betaproteobacteria	Burkholderiales	betIII	betIII-A	betIII-A1	heterotroph	organo	N/A	-0.17	0.53	0.29	3.04	0.00	0.50
Otu00020	Bacteroidetes	Flavobacteria	Flavobacteriales	bacII	bacII-A	unclassified	heterotroph	organo	Livermore et al., 2014	-0.55	0.03	0.61	0.42	0.13	1.53
Otu00021	Verrucomicrobia	OPB35_soil_group	unclassified	unclassified	unclassified	NA	heterotroph	organo	N/A	-0.62	0.01	1.85	2.41	0.00	0.88
Otu00022	Verrucomicrobia	Verrucomicrobiae	Verrucomicrobiales	Verrucomicrobiaceae	unclassified	NA	heterotroph	organo	N/A	-0.65	0.07	0.63	-4.01	0.00	4.17
Otu00024	Alphaproteobacteria	Alphaproteobacteria	Sphingomonadales	alfIV	alfIV-B	Pyxis	heterotroph	organo	Livermore et al., 2014	0.00	0.99	0.72	2.41	0.00	1.66
Otu00025	Betaproteobacteria	Betaproteobacteria	Burkholderiales	betVII	betVII-B	betVII-B1	heterotroph	organo	Hornung et al., 2013	-	-	-	1.69	0.02	0.18
Otu00028	Verrucomicrobia	Spartobacteria	Spartobacteriales	Spartobacteriaceae	CandidatusXiphinematobacter	verI-B	heterotroph	organo	Herlemann et al., 2013	0.22	0.45	2.10	2.20	0.00	1.06
Otu00029	Bacteroidetes	Flavobacteria	Flavobacteriales	bacV	unclassified	unclassified	heterotroph	organo	Newton et al., 2011	-0.60	0.02	1.61	0.71	0.00	1.49
Otu00030	Planctomycetes	Phycisphaerae	Phycisphaerales	Phycisphaeraceae	CL500-3	NA	heterotroph	organo	Fuerst and Sagulenko, 2011; Orsi et	-1.39	0.00	0.12	-4.06	0.00	0.17

									al., 2016						
Otu00031	Actinobacteria	Actinobacteria	Actinomycetales	acI	acI-A	acI-A4	heterotroph	organo/photo(RHO)	Ghylin et al., 2014	0.59	0.00	0.90	4.46	0.00	0.67
Otu00032	Bacteroidetes	Flavobacteria	Flavobacteriales	bacII	bacII-A	Flavo-A3	heterotroph	organo	Livermore et al., 2014	-0.36	0.05	0.65	0.08	0.75	0.79
Otu00033	Actinobacteria	Actinobacteria	Actinomycetales	acI	acI-A	Phila	heterotroph	organo/photo(RHO)	Ghylin et al., 2014	0.33	0.07	0.40	3.48	0.00	0.13
Otu00034	Cyanobacteria	Cyanobacteria	SubsectionI	FamilyI	Synechococcus	NA	autotroph	photo(CHL)	Boone and Castenholz, 2012	-0.74	0.05	0.87	0.56	0.10	0.44
Otu00035	Bacteroidetes	Flavobacteria	Flavobacteriales	bacV	unclassified	unclassified	heterotroph	organo	Newton et al., 2011	-	-	-	1.51	0.03	0.82
Otu00036	Bacteroidetes	Sphingobacteria	Sphingobacteriales	bacI	unclassified	unclassified	heterotroph	organo	Newton et al., 2011; Woodhouse et al., 2016	-0.13	0.57	0.38	1.83	0.00	1.72
Otu00037	Chloroflexi	Anaerolineae	Anaerolineales	Anaerolineaceae	unclassified	NA	heterotroph	organo/photo(RHO)	Denef et al., 2016	0.35	0.03	3.48	3.82	0.00	1.05
Otu00039	Bacteroidetes	Sphingobacteria	Sphingobacteriales	bacI	bacI-A	unclassified	heterotroph	organo	Newton et al., 2011; Woodhouse et al., 2016	-0.46	0.20	0.33	2.03	0.00	2.01
Otu00040	Actinobacteria	Actinobacteria	Acidimicrobiales	acIV	acIV-A	Iluma-A2	heterotroph	organo/photo(RHO)	Hugerth et al., 2015	0.15	0.55	3.72	4.25	0.00	0.45
Otu00041	Actinobacteria	Actinobacteria	Acidimicrobiales	acIV	acIV-A	Iluma-A1	heterotroph	organo/photo(RHO)	Hugerth et al., 2015	0.41	0.01	1.11	4.32	0.00	0.30
Otu00042	Actinobacteria	Actinobacteria	Actinomycetales	acI	acI-C	acI-C2	heterotroph	organo/photo(RHO)	Newton et al., 2011	0.51	0.00	0.72	5.02	0.00	0.47
Otu00043	Planctomycetes	Phycisphaerae	Phycisphaerales	Phycisphaeraceae	CL500-3	NA	heterotroph	organo	Fuerst and Sagulenko, 2011; Orsi et al., 2016	-0.71	0.01	0.24	-2.17	0.00	0.75
Otu00044	Chloroflexi	SL56_marine_group	unclassified	unclassified	unclassified	NA	heterotroph	organo	N/A	0.73	0.04	1.76	4.02	0.00	0.31
Otu00047	Actinobacteria	Actinobacteria	Actinomycetales	Luna1	Luna1-A	unclassified	heterotroph	organo/photo(RHO)	Newton et al., 2011	0.43	0.09	0.40	3.59	0.00	0.18

Otu00048	Bacteroidetes	Flavobacteria	Flavobacteriales	bacII	bacII-A	Flavo-A1	heterotroph	organo	Livermore et al., 2014	-0.73	0.00	0.45	-0.54	0.16	0.70
Otu00049	Alphaproteobacteria	Alphaproteobacteria	Sphingomonadales	alfIV	unclassified	unclassified	heterotroph	organo	Livermore et al., 2014	-	-	-	-1.11	0.24	0.20
Otu00050	Proteobacteria	unclassified	unclassified	unclassified	unclassified	NA	heterotroph	organo	N/A	0.12	0.69	1.65	1.67	0.00	1.80
Otu00051	Actinobacteria	Actinobacteria	Actinomycetales	acSTL	acSTL-A	acSTL-A1	heterotroph	organo/photo(RHO)	Martinez-Garcia et al., 2012	0.71	0.00	0.98	3.88	0.00	0.45
Otu00052	Verrucomicrobia	Opitutae	Opitutales	Opitutaceae	unclassified	unclassified	heterotroph	organo(C1)	Kalyuzhnaya et al., 2008	-0.13	0.69	1.47	2.30	0.00	0.23
Otu00053	Actinobacteria	Actinobacteria	Actinomycetales	acI	acI-A	acI-A5	heterotroph	organo/photo(RHO)	Ghylin et al., 2014	0.40	0.02	0.50	4.80	0.00	0.37
Otu00055	Alphaproteobacteria	Alphaproteobacteria	Caulobacterales	Hyphomonadaceae	Hirschia	NA	heterotroph	organo	Chertkov et al., 2011	-	-	-	-3.78	0.00	0.19
Otu00056	Verrucomicrobia	Opitutae	Opitutales	Opitutaceae	unclassified	unclassified	heterotroph	organo(C1)	Kalyuzhnaya et al., 2008	-0.50	0.05	0.27	2.88	0.00	0.19
Otu00057	Actinobacteria	Actinobacteria	Actinomycetales	acTH1	acTH1-A	acTH1-A1	heterotroph	organo/photo(RHO)	Newton et al., 2011	0.54	0.04	0.87	3.68	0.00	0.36
Otu00058	Cyanobacteria	Cyanobacteria	SubsectionIV	FamilyI	Anabaena	NA	autotroph	photo(CHL)	Boone and Castenholz, 2012	-	-	-	-6.88	0.00	0.15
Otu00059	Alphaproteobacteria	Alphaproteobacteria	Rhizobiales	alfI	alfI-A	alfI-A1	heterotroph	organo	N/A	0.49	0.00	1.10	5.43	0.00	0.68
Otu00061	Betaproteobacteria	Betaproteobacteria	Burkholderiales	betI	betI-A	unclassified	heterotroph	organo	Newton et al., 2011	0.90	0.00	0.18	3.06	0.00	0.12
Otu00062	Bacteroidetes	Flavobacteria	Flavobacteriales	bacII	bacII-A	unclassified	heterotroph	organo	Livermore et al., 2014	-0.22	0.67	0.11	-0.99	0.09	0.12
Otu00064	Bacteroidetes	Flavobacteriia	Flavobacteriales	Cryomorphaceae	Owenweeksia	NA	heterotroph	organo	Boone and Castenholz, 2012	-0.35	0.14	0.23	2.69	0.00	1.02
Otu00065	Deltaproteobacteria	Deltaproteobacteria	Bdellovibrionales	Bdellovibrionaceae	OM27_clade	NA	heterotroph	organo	Orsi et al., 2016	-0.46	0.07	0.29	-4.28	0.00	0.35
Otu00066	Verrucomicrobia	Opitutae	Opitutales	Opitutaceae	unclassified	unclassified	heterotroph	organo(C1)	Kalyuzhnaya et al., 2008	-0.71	0.04	0.21	1.63	0.00	0.16
Otu00067	Bacteroidetes	unclassified	unclassified	unclassified	unclassified	NA	heterotroph	organo	N/A	-	-	-	-4.63	0.00	0.15

Otu00068	Bacteroidetes	Sphingobacteriia	Sphingobacteriales	env.OPS_17	unclassified	NA	heterotroph	organo	Salcher et al., 2013	-0.19	0.56	0.17	-2.72	0.00	0.26
Otu00070	Armatimonadetes	Armatimonadia	Armatimonadales	Armatimonadaceae	Armatimonas	NA	heterotroph	organo	Tamaki et al., 2011	-1.11	0.00	0.30	-1.52	0.01	0.26
Otu00073	Verrucomicrobia	Opitutae	Opitutales	Opitutaceae	unclassified	unclassified	heterotroph	organo(C1)	Kalyuzhnaya et al., 2008	-0.63	0.01	0.29	3.76	0.00	0.30
Otu00075	Alphaproteobacteria	Alphaproteobacteria	Caulobacterales	alfII	alfII-A	Brev	heterotroph	organo	Livermore et al., 2014	-0.53	0.12	0.15	-0.62	0.17	0.25
Otu00078	Bacteroidetes	Flavobacteria	Flavobacteriales	bacV	unclassified	unclassified	heterotroph	organo	Newton et al., 2011	-0.33	0.45	0.43	-1.14	0.00	0.61
Otu00081	Bacteroidetes	Sphingobacteria	Sphingobacteriales	bacI	unclassified	unclassified	heterotroph	organo	Newton et al., 2011; Woodhouse et al., 2016	-0.06	0.84	0.35	1.07	0.07	0.48
Otu00082	Bacteroidetes	Flavobacteria	Flavobacteriales	bacII	bacII-A	Flavo-A3	heterotroph	organo	Livermore et al., 2014	-0.69	0.11	0.13	-2.14	0.00	0.24
Otu00084	Planctomycetes	Phycisphaerae	Phycisphaerales	Phycisphaeraceae	CL500-3	NA	heterotroph	organo	Fuerst and Sagulenko, 2011; Orsi et al., 2016	-0.82	0.01	0.28	-0.66	0.17	0.64
Otu00086	Bacteroidetes	Cytophagia	Cytophagales	unclassified	unclassified	NA	heterotroph	organo	Sheu et al., 2009	-	-	-	-2.74	0.00	0.24
Otu00089	Bacteroidetes	Flavobacteria	Flavobacteriales	bacII	bacII-A	Flavo-A2	heterotroph	organo	Livermore et al., 2014	-0.77	0.00	0.44	-0.08	0.84	0.38
Otu00091	Verrucomicrobia	Methylacidiphilae	Methylacidiphilales	LD19	unclassified	unclassified	autotroph	organo(C1)	Sharp et al., 2012	0.02	0.94	0.19	-	-	-
Otu00093	Bacteroidetes	Sphingobacteriia	Sphingobacteriales	NS11-12_marine_group	unclassified	NA	heterotroph	organo	Salcher et al., 2013	-0.72	0.01	0.26	0.95	0.01	0.36
Otu00095	Actinobacteria	Actinobacteria	Acidimicrobiales	acIV	acIV-C	Iluma-C1	heterotroph	organo/photo(RHO)	Hugerth et al., 2015	0.48	0.08	0.51	-	-	-
Otu00098	Gammaproteobacteria	Gammaproteobacteria	Pseudomondales	gamIII	gamIII-A	Acin	heterotroph	organo	N/A	-	-	-	-3.05	0.00	0.27
Otu00099	Bacteroidetes	Flavobacteria	Flavobacteriales	bacV	unclassified	unclassified	heterotroph	organo	Newton et al., 2011	-0.30	0.50	0.24	1.60	0.00	0.29
Otu00105	Actinobacteria	Actinobacteria	Acidimicrobiales	acIV	acIV-B	Iluma-B1	heterotroph	organo/photo(RHO)	Hugerth et al., 2015	-0.19	0.64	0.59	2.59	0.00	0.14

Otu00108	Bacteroidetes	Sphingobacteria	Sphingobacteriales	bacI	bacI-A	unclassified	heterotroph	organo	Newton et al., 2011; Woodhouse et al., 2016	0.38	0.33	0.17	2.00	0.02	0.21
Otu00109	Bacteroidetes	Sphingobacteria	Sphingobacteriales	bacVI	unclassified	unclassified	heterotroph	organo	Salcher et al., 2013	-	-	-	-0.65	0.16	0.19
Otu00113	Bacteroidetes	Sphingobacteria	Sphingobacteriales	bacVI	unclassified	unclassified	heterotroph	organo	Salcher et al., 2013	-0.60	0.01	0.61	1.62	0.00	0.53
Otu00114	Actinobacteria	Actinobacteria	Actinomycetales	acI	unclassified	unclassified	heterotroph	organo/photo(RHO)	Ghylin et al., 2014	0.30	0.06	0.82	6.43	0.00	0.19
Otu00119	Bacteroidetes	Sphingobacteria	Sphingobacteriales	bacVI	bacVI-B	Pedo	heterotroph	organo	Salcher et al., 2013; Woodhouse et al., 2016	-0.14	0.63	0.13	3.27	0.00	0.15
Otu00121	Actinobacteria	Actinobacteria	Acidimicrobiales	acIV	acIV-A	Iluma-A2	heterotroph	organo/photo(RHO)	Hugerth et al., 2015	0.17	0.57	0.72	-	-	-
Otu00124	Deltaproteobacteria	Deltaproteobacteria	Bdellovibrionales	Bdellovibrionaceae	OM27_clade	NA	heterotroph	organo	Orsi et al., 2016	-0.67	0.10	0.18	-2.87	0.00	0.56
Otu00126	Bacteroidetes	Flavobacteria	Flavobacteriales	bacII	bacII-A	unclassified	heterotroph	organo	Livermore et al., 2014	-0.17	0.67	0.13	-0.71	0.15	0.21
Otu00128	Actinobacteria	Actinobacteria	Acidimicrobiales	acIV	acIV-D	Iamia	heterotroph	organo/photo(RHO)	Hugerth et al., 2015	0.27	0.44	0.20	-	-	-
Otu00132	Verrucomicrobia	Spartobacteria	Chthoniobacterales	zEL20	unclassified	NA	heterotroph	organo	Sangwan et al., 2004	-0.85	0.00	1.45	2.18	0.00	0.50
Otu00147	Bacteroidetes	Sphingobacteria	Sphingobacteriales	bacVI	unclassified	unclassified	heterotroph	organo	Salcher et al., 2013	-	-	-	-4.23	0.00	0.14
Otu00149	Alphaproteobacteria	Alphaproteobacteria	Rhodobacterales	Rhodobacteraceae	unclassified	NA	heterotroph	organo/photo(BCHL)	Boone and Castenholz, 2012	0.21	0.67	0.35	-	-	-
Otu00152	Bacteroidetes	Sphingobacteria	Sphingobacteriales	bacI	unclassified	unclassified	heterotroph	organo	Newton et al., 2011; Woodhouse et al., 2016	-	-	-	1.13	0.01	0.12
Otu00162	Actinobacteria	Actinobacteria	Actinomycetales	acI	acI-C	acI-C1	heterotroph	organo/photo(RHO)	Newton et al., 2011	0.38	0.15	0.33	4.84	0.00	0.12
Otu00168	Verrucomicrobia	Opitutae	Opitutales	Opitutaceae	unclassified	unclassified	heterotroph	organo(C1)	Kalyuzhnaya et al.,	-0.30	0.21	0.23	-	-	-

									2008						
Otu00169	Gammaproteobacteria	Gammaproteobacteria	Oceanospirillales	Oceanospirillaceae	Pseudospirillum	NA	heterotroph	organo/photo(RHO)	Boone and Castenholz, 2012	-0.72	0.10	0.12	-	-	-
Otu00195	Planctomycetes	Phycisphaerae	Phycisphaerales	Phycisphaeraceae	CL500-3	NA	heterotroph	organo	Fuerst and Sagulenko, 2011; Orsi et al., 2016	-0.78	0.04	0.26	-1.20	0.04	0.44
Otu00196	Verrucomicrobia	Methylacidiphilae	Methylacidiphilales	LD19	unclassified	unclassified	autotroph	organo(C1)	Sharp et al., 2012	-0.12	0.53	0.57	2.24	0.00	0.25
Otu00229	Bacteroidetes	Sphingobacteriia	Sphingobacteriales	NS11-12_marine_group	unclassified	NA	heterotroph	organo	Salcher et al., 2013	-	-	-	0.83	0.01	0.15
Otu00251	Bacteroidetes	Sphingobacteriia	Sphingobacteriales	env.OPS_17	unclassified	NA	heterotroph	organo	Salcher et al., 2013	-0.26	0.55	0.25	0.68	0.15	0.43
Otu00293	Bacteroidetes	Flavobacteria	Flavobacteriales	bacV	unclassified	unclassified	heterotroph	organo	Newton et al., 2011	0.09	0.84	0.20	0.26	0.62	0.15
Otu00334	Planctomycetes	Planctomycetacia	Planctomycetales	Planctomycetaceae	unclassified	NA	heterotroph	organo	Fuerst and Sagulenko, 2011	-0.85	0.01	0.78	2.14	0.01	0.47
Otu00375	Planctomycetes	Phycisphaerae	Phycisphaerales	Phycisphaeraceae	CL500-3	NA	heterotroph	organo	Fuerst and Sagulenko, 2011; Orsi et al., 2016	-0.71	0.05	0.17	-0.99	0.04	0.21
Otu00465	Actinobacteria	Thermoleophilia	unclassified	unclassified	unclassified	NA	heterotroph	organo	N/A	0.28	0.27	0.19	-	-	-
Otu00512	Actinobacteria	Actinobacteria	Acidimicrobiales	acIV	unclassified	unclassified	heterotroph	organo/photo(RHO)	Hugerth et al., 2015	0.25	0.45	0.15	-	-	-
